# A self-binding immune complex vaccine elicits strong neutralizing responses against herpes simplex virus in mice

**DOI:** 10.3389/fimmu.2023.1085911

**Published:** 2023-05-02

**Authors:** Andrew G. Diamos, Mary D. Pardhe, Melissa H. Bergeman, Aigerim S. Kamzina, Michelle P. DiPalma, Sara Aman, Artemio Chaves, Kenneth Lowe, Jacquelyn Kilbourne, Ian B. Hogue, Hugh S. Mason

**Affiliations:** Center for Immunotherapy, Vaccines, and Virotherapy, Biodesign Institute at Arizona State University (ASU), School of Life Sciences, Arizona State University, Tempe, AZ, United States

**Keywords:** herpes simplex virus (HSV), vaccine, immune complex (IC), plant-made, complement receptor c1q, neutralizing antibodies, glycoprotein D (gD), IgG fusion

## Abstract

**Introduction:**

It has been known for over half a century that mixing an antigen with its cognate antibody in an immune complex (IC) can enhance antigen immunogenicity. However, many ICs produce inconsistent immune responses, and the use of ICs in the development new vaccines has been limited despite the otherwise widespread success of antibody-based therapeutics. To address this problem, we designed a self-binding recombinant immune complex (RIC) vaccine which mimics the larger ICs generated during natural infection.

**Materials and methods:**

In this study, we created two novel vaccine candidates: 1) a traditional IC targeting herpes simplex virus 2 (HSV-2) by mixing glycoprotein D (gD) with a neutralizing antibody (gD-IC); and 2) an RIC consisting of gD fused to an immunoglobulin heavy chain and then tagged with its own binding site, allowing self-binding (gD-RIC). We characterized the complex size and immune receptor binding characteristics in vitro for each preparation. Then, the in vivo immunogenicity and virus neutralization of each vaccine were compared in mice.

**Results:**

gD-RIC formed larger complexes which enhanced C1q receptor binding 25-fold compared to gD-IC. After immunization of mice, gD-RIC elicited up to 1,000-fold higher gD-specific antibody titers compared to traditional IC, reaching endpoint titers of 1:500,000 after two doses without adjuvant. The RIC construct also elicited stronger virus-specific neutralization against HSV-2, as well as stronger cross-neutralization against HSV-1, although the proportion of neutralizing antibodies to total antibodies was somewhat reduced in the RIC group.

**Discussion:**

This work demonstrates that the RIC system overcomes many of the pitfalls of traditional IC, providing potent immune responses against HSV-2 gD. Based on these findings, further improvements to the RIC system are discussed. RIC have now been shown to be capable of inducing potent immune responses to a variety of viral antigens, underscoring their broad potential as a vaccine platform.

## Highlights

- Self-binding immune complexes increase immune receptor C1q binding 25-fold over traditional immune complexes.
- Antigen-specific antibody titers increased by up to 1,000-fold using self-binding immune complexes compared to traditional immune complexes.
- Improved neutralizing and cross-neutralizing antibodies against HSV-2 and HSV-1.

## 1 Introduction

Antibodies are one of the most widely produced therapeutic agents, comprising the largest share of the global biopharmaceutical market. In 2021, the one-hundredth antibody therapy was approved by the FDA (1). While antibodies by themselves are highly useful, it is becoming increasingly common to fuse antibodies to other proteins of interest to imbue them with desirable properties. Fusion to IgG antibody often provides enhanced solubility and stability of the fusion partner due to the inherent stability of IgG molecules and allows simple and highly efficient purification via protein A/G affinity chromatography (2). Additionally, IgG fusions may have extended serum half-life, as IgG are protected from degradation in endosomes due to their ability to bind neonatal Fc receptor (FcRn) (3).

Though less explored, IgG fusion molecules also have additional properties uniquely suited to the creation of potent vaccines. Antibody-antigen complexes are directly taken up by antigen-presenting cells such as dendritic cells, macrophages, and B cells via the interactions of the IgG Fc with FcRn receptors (3–5), complement receptors (6, 7), and Fcγ receptors (8). However, not all antibody-antigen molecules are potent immunogens. When repetitive antigens are bound by antibody, they form larger immune complexes (ICs) which are more potent activators of immune receptors than monomeric antibody-bound antigen. For instance, the complement receptor C1q requires simultaneous engagement of its six head regions with six IgG Fc regions, and thus monomeric antibody poorly activates complement, whereas multimeric ICs potently activate complement (9, 10). Complement activation leads to iC3b coating of the ICs as well as release of complement anaphylatoxins, resulting in the recruitment of immune cells to the site of vaccination, deposition of complexed antigen onto follicular dendritic cells, and subsequent stimulation of both B cell and T cell immunity (11, 12). In a similar fashion, larger ICs, but not monomeric antibody-bound antigen, can efficiently cross-link low affinity Fcγ receptors, leading to further enhanced uptake and stimulation by antigen presenting cells (13).

To harness the benefits of IgG fusions, several vaccine platforms have been designed. Perhaps the simplest method is to simply fuse an antigen of interest to the IgG Fc (2, 14). The most successful example of this strategy is a SARS-CoV-2 vaccine consisting of interferon-*α*, the pan HLA-DR-binding epitope (PADRE), and the SARS-CoV-2 spike receptor binding domain fused to IgG1 Fc. Compared to immunization with the receptor binding domain alone, the Fc fusion was found in higher abundance in lymph nodes, and safely generated strong Th1, Th2, CD8+ and neutralizing antibody responses in rhesus macaques (15) and in humans (16, 17). Intriguingly, a vaccine comprising herpes simplex virus (HSV) 2 glycoprotein D (gD) fused to an IgG Fc could be efficiently administered mucosally, as FcRn receptors mediate uptake of IgG across mucosal epithelial surfaces (18). In a different strategy, mixing specific antibodies with antigens to form an immune complex (IC) has been used to focus immune responses towards favorable antigenic sites on tick-borne encephalitis virus (19) and HIV (20). Vaccination with sialylated ICs targeting influenza hemagglutinin was found to improve the breadth and potency of anti-influenza antibodies by selecting for high affinity B cells (21). These studies underscore the unique benefits of IgG fusion vaccines.

While some Fc fusions may spontaneously form multimeric structures capable of engaging complement and low affinity Fcγ immune receptors (10, 22), their stability and antigenicity appear to be strongly dependent on the characteristics of the fusion partner, such as whether the fused antigen forms multimers (23). Therefore, strategies have been developed to generate consistently immunogenic antigen-antibody structures. In one strategy, antigen was delivered on an IgG1 containing the IgM tailpiece and coexpressed with the J chain, forming pentameric and hexameric molecules (24). These constructs efficiently engaged C1q and low affinity Fcγ receptors, providing robust B cell and T cell immunity in mice and in human adenotonsillar tissue against the dengue virus envelope protein (25). This strategy was also successful when used to orally deliver an antigen derived from porcine epidemic diarrhea virus (26).

We designed a vaccine platform consisting of self-multimerizing ICs capable of forming highly immunogenic clusters with antigens of interest, called recombinant immune complexes (RICs) (27–30). In this system, the antigen is fused to the well-characterized mAb 6D8 which has been tagged with its own binding site, allowing multiple antigen-antibody molecules to bind to each other to form larger complexes. In the present study, to investigate the key differences between traditional IC and the RIC system, we compared the immunogenic properties of herpes simplex virus 2 (HSV-2) glycoprotein D (gD) delivered either via traditional monomeric IC or via self-interacting multimeric RIC.

## 2 Results

### 2.1 Production and characterization gD-IC and gD-RIC

**Figure 1:**
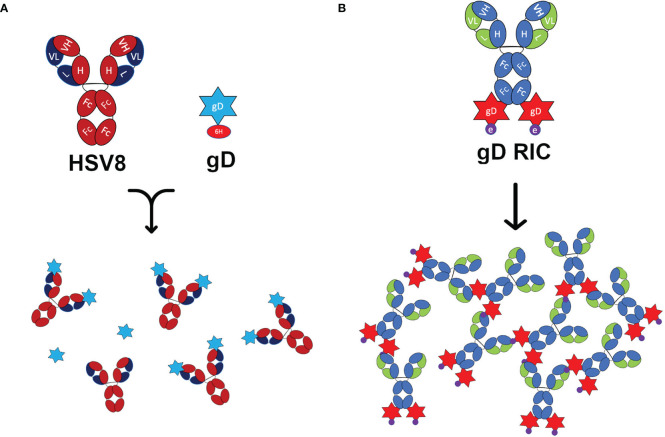
Schematic representation of the constructs used in this study. **(A)** Representations of the human IgG1 monoclonal antibody HSV8 and 6-histidine tagged glycoprotein D (gD) from HSV-2. When mixed, HSV8 can bind up to two gD molecules forming an immune complex (IC). **(B)** The recombinant immune complex (RIC) construct contains the 6D8 monoclonal antibody with C-terminal fusion to gD connected via linker with the epitope tag “e.” This construct forms self-binding clusters via the interaction of the 6D8 variable regions with the epitope tag “e” on another gD-RIC molecule. This interaction results in the formation of larger immune complexes.

**Figure 2:**
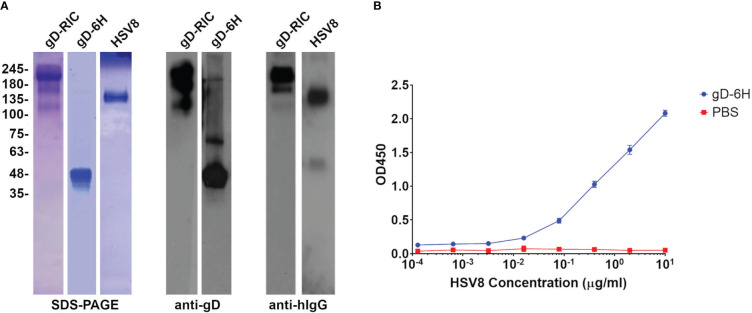
Purification of gD constructs and antibody binding assay. **(A)** SDS-PAGE and Western blots of the plant-made purified gD constructs. The gD samples were run under reducing conditions, whereas the antibody and RIC constructs were run under nonreducing conditions. The Western blots were probed with either HSV8 (for gD-6H) or H170 (for gD-RIC) to recognize gD, or with an anti-human IgG (H+L) HRP-conjugated antibody. **(B)** An ELISA was performed to test the binding between plant-made gD and HSV8. Purified gD was bound to the plate, probed with various concentrations of HSV8, and detected with anti-human IgG-HRP conjugate. Points represent mean OD450 ± standard deviation from two replicates.

To create a traditional IC targeting HSV, the neutralizing mAb HSV8, which recognizes a conformational epitope in gD (31), was expressed in plants and purified along with the soluble ectodomain from HSV-2 gD (amino acids 26-331) containing a 6-histidine tag (**Figure 1**, constructs “HSV8” and “gD”). By SDS-PAGE, HSV8 formed bands at ~150 kDa, while gD formed bands around ~48 kDa, which is expected for glycosylated gD (**Figure 2A**). HSV8 readily detected gD via nonreducing western blot (**Figure 2A**) but not by reducing western blot (data not shown). To further verify the binding of plant-made HSV8 and gD in solution, an ELISA was performed. HSV8 showed robust binding to gD at concentrations as low as 3 nM (**Figure 2B**), indicating that IC formation occurs readily in solution.

Next, an RIC vector targeting HSV was created by inserting the DNA sequence encoding HSV-2 gD (amino acids 26-331) at the C-terminus of the heavy chain of the human IgG1 6D8 tagged with its own binding site (**Figure 1**, construct “gD-RIC”). When purified gD-RIC was probed with HSV8 or anti-human IgG by western blot, a band was seen around the expected size of ~218 kDa for fully formed gD-RIC containing both human IgG1 and gD antigen (**Figure 2**).

### 2.2 gD-RIC form larger complexes with improved immune receptor binding compared to gD-IC

**Figure 3:**
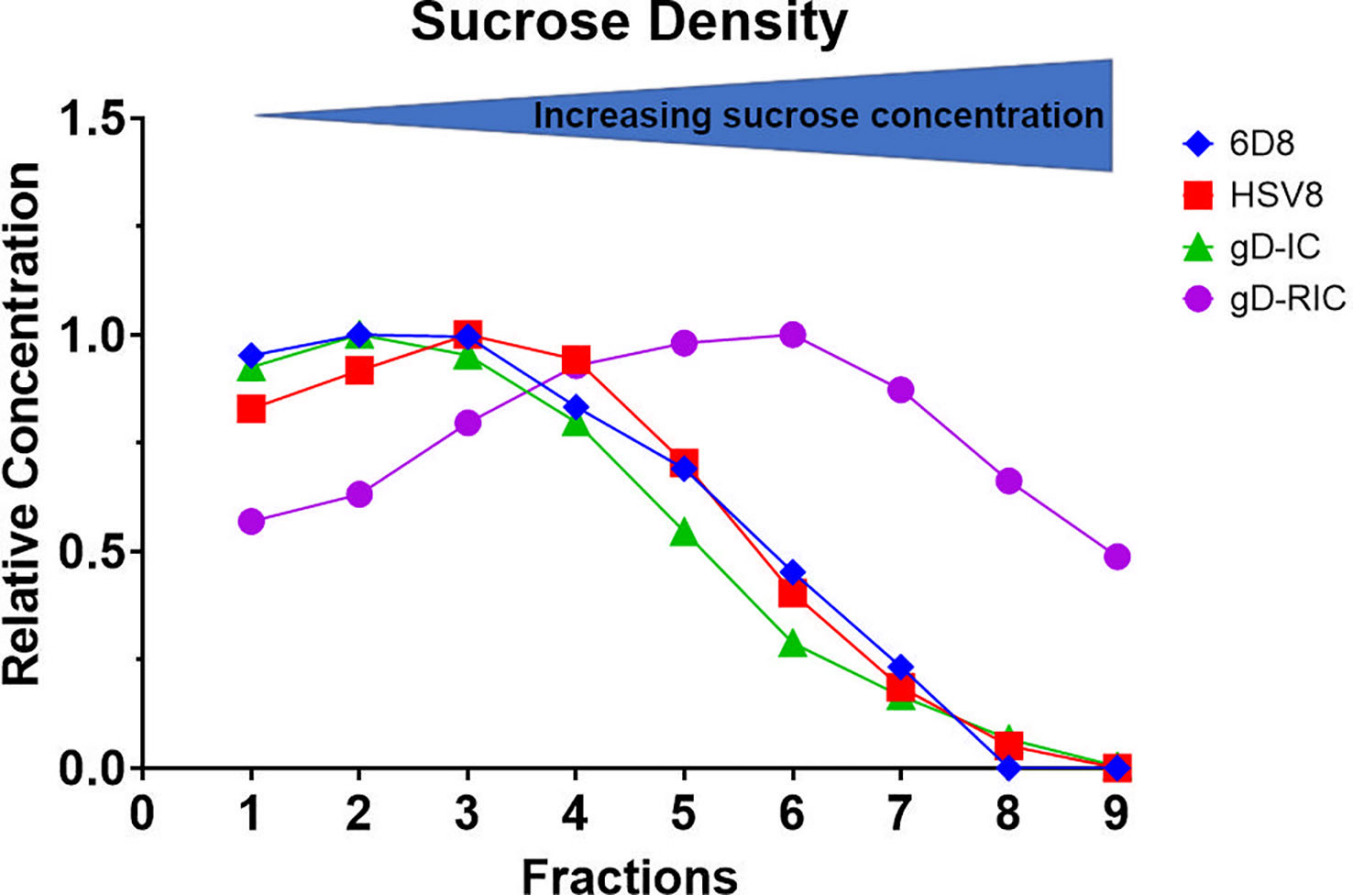
Sucrose gradient density profile of gD-IC and gD-RIC. Using HSV8 and 6D8 mAbs as controls, gD-IC and gD-RIC were separated by sucrose gradient sedimentation using 5/10/15/20/25% discontinuous sucrose layers. The protein concentration of each fraction was analyzed by spectrophotometry and representative results from three independent experiments are shown; direction of sedimentation is left to right. The peak concentration was arbitrarily assigned a value of 1.

To determine whether gD-RIC form larger complexes than gD-IC, both constructs were analyzed by sucrose gradient sedimentation using the monomeric antibodies 6D8 and HSV8 as controls. Since gD-RIC contains potentially two gD molecules per antibody molecule, gD-IC were also prepared by preincubating HSV8 with gD at a molar ratio of 1:2. Whereas gD-IC did not display notable differences in density compared to the controls, gD-RIC was found to sediment substantially faster, forming a broad peak consistent with the formation of large heterogenous complexes (**Figure 3**).

**Figure 4:**
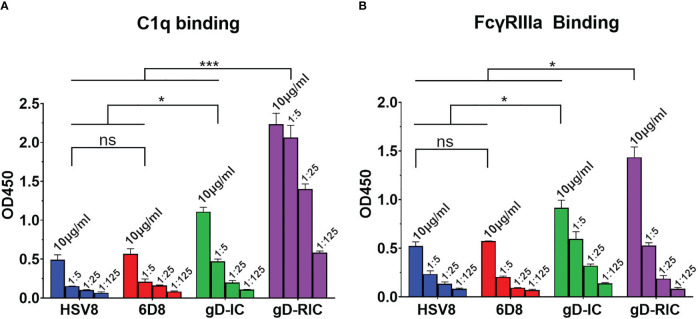
C1q and FcyRIIIa binding. Immune receptor binding ELISA of gD-IC and gD-RIC. ELISA plates were coated with 15 μg/ml **(A)** human C1q and **(B)** human FcyRIIIa and incubated with 5-fold serial dilutions of each construct starting at 10 μg/ml, using HSV8 and 6D8 mAbs as monomeric controls. Constructs were detected using polyclonal goat anti-human IgG-HRP. Mean OD450 values from three samples are shown ± standard error. Three stars (***) indicates p < 0.001 and one star (*) indicates p < 0.05 between the indicated columns using one-way ANOVA with Tukey’s post-test for multiple comparisons. ns, not significant.

Complement receptor C1q preferentially binds multimeric IgG, with hexamer or larger complexes having the strongest binding (9). All antibody constructs were expressed in plants silenced for the plant-specific glycans fucose and xylose, which has been shown to improve antibody immune receptor binding (29, 32). The mAbs 6D8 and HSV8 showed minimal binding to C1q, while gD-HSV8 IC showed somewhat improved binding (**Figure 4A**, p < 0.05). By stark contrast, gD-RIC showed a 25-fold increase in C1q binding compared to gD-IC (**Figure 4A**, p < 0.001, compare gD-RIC column 3, representing 25-fold dilution, to gD-IC column 1, undiluted**).** Low affinity receptor FcγRIIIa binding was also measured by ELISA. Both IC and RIC (**Figure 4B**, p < 0.05) displayed improved FcγRIIIa binding compared to monomeric controls, with RIC showing the highest binding (**Figure 4B**, p < 0.05).

### 2.3 gD-RIC are highly immunogenic in mice compared to gD-IC

**Figure 5:**
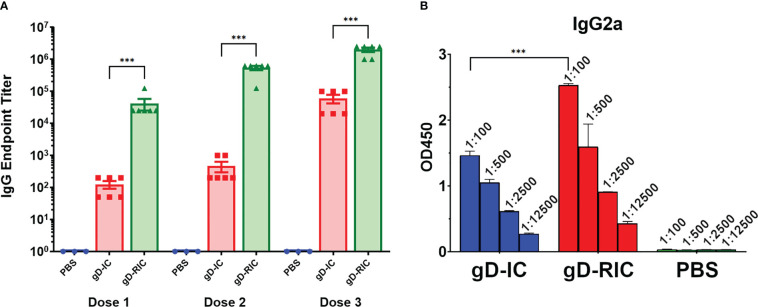
Mouse immunization and serum titers. BALB/c mice (6 per group) were immunized three times three weeks apart subcutaneously with a dose that would deliver 4 μg gD for each construct or with PBS as a control. Mouse serum samples was collected on days 28, 56, and 86 for doses 1, 2, and 3 respectively. **(A)** Serially diluted mouse serum was analyzed for total gD-specific IgG production by ELISA. The endpoint was taken as the reciprocal of the greatest dilution that gave an OD_450_ reading at least twice the background. Three stars (***) indicates p < 0.001 by one-way ANOVA using Tukey’s post-test comparing the indicated columns. **(B)** Dose 3 mouse serum samples were serially diluted 5-fold starting at a 1:100 dilution and analyzed for IgG2a production by ELISA. Mean OD450 values from three samples are shown ± standard error. Three stars (***) indicates p < 0.001 by one-way ANOVA using Tukey’s post-test comparing the indicated columns.

**Figure 6:**
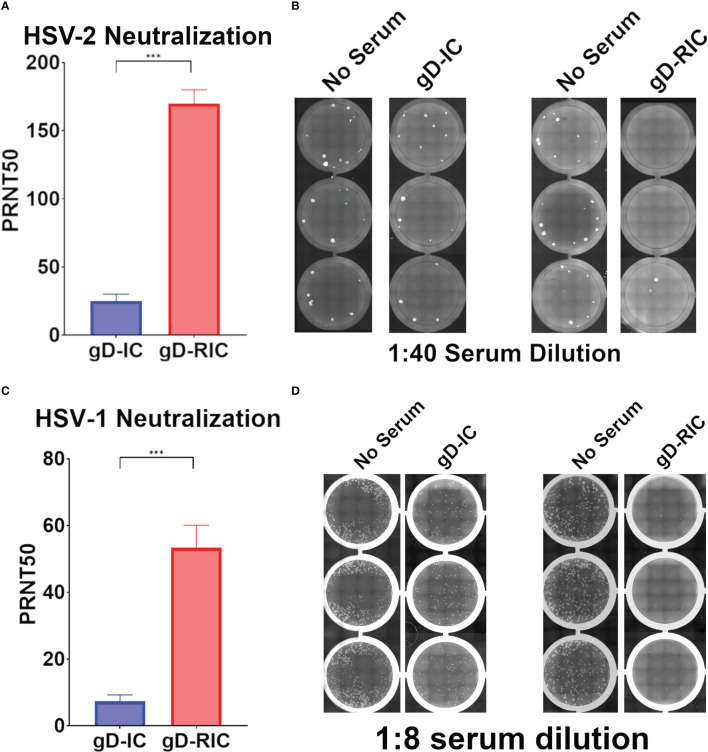
Neutralization of HSV-2. Plaque reduction neutralization 50 (PRNT50) assays of **(A, B)** HSV-2 or **(C, D)** HSV-1 were performed using serially diluted mouse serum samples from dose 3. Data represent the mean and standard error from four samples for HSV-2, or six samples for HSV-1. Three stars (***) indicates p < 0.001 by student’s *t*-test. Representative images showing virus plaques from 3 replicate serum-containing wells and 3 replicate control wells are shown for both HSV-2 and HSV-1.

To test the *in vivo* immunogenicity of each construct, BALB/c mice were immunized three times with 4 μg gD delivered either as gD-IC or gD-RIC without adjuvant. After each dose, the resulting mouse serum was analyzed for gD-specific antibody titers by ELISA. Strikingly, mice immunized with gD-RIC produced titers that exceeded those of the IC-immunized mice by 332-fold, 1162-fold, and 33-fold after doses 1, 2 and 3 respectively (**Figure 5A**, p < 0.001). gD-RIC produced 5-fold higher levels of IgG2a antibodies compared to gD-IC (**Figure 5B**, p < 0.05). Finally, the neutralization of herpes simplex virus 2 (HSV-2) using serum from gD-IC or gD-RIC immunized mice was compared. gD-RIC serum neutralized HSV-2 significantly more than gD-IC serum (**Figures 6A, B**, p < 0.001). To assess the cross-neutralizing potential of the immune serum, neutralization with HSV-1 was also performed. gD-RIC serum cross-neutralized HSV-1 significantly more than gD-IC serum (**Figures 6C, D**, p < 0.001).

## 3 Discussion

HSV infection of neonates has as high as a 50% chance of developing disseminated disease or encephalitis, with current drug options still leaving approximately 70% of neonates with long-term neurological sequelae (33). Neonatal infection often occurs (55%) if the mother becomes infected for the first time during pregnancy, whereas there is minimal risk if the mother has previously been infected (<1%), likely due to the transfer of maternal antibodies (34–36). Building on these findings, it has recently been shown that vaccination of the mother can also prevent neonatal HSV infection in a mouse model (37). These results underscore the need for safe, effective, and cheap HSV vaccines. In the present study, we show that a self-binding antibody complex formed with gD (gD-RIC) induces robust gD-specific antibody production and neutralizes both HSV-1 and HSV-2, strongly outperforming a traditional IC composed of gD simply mixed with a neutralizing antibody (gD-IC) (**Figures 5**, **6**).

Past research has repeatedly demonstrated that the delivery of IC composed of an antigen mixed with antisera can enhance the immune response towards a given antigen compared to antigen delivery alone (38). However, many studies have found inconsistent results, including reduced immunogenicity of IC vaccines and, despite being studied for over half a century, IC-based therapeutics have failed to produce a single FDA-approved vaccine (39, 40). In this study, both vaccine preparations contain equivalent total amounts of both antibody and antigen delivered in the same ratio: approximately one antibody molecule per two gD molecules. Nevertheless, gD-RIC produced strikingly higher immune responses, up to 1,000-fold higher antigen-specific antibody titers after 2 doses (**Figure 5A**). The immunogenicity of a given IC depends strongly on the individual properties of each antibody and the characteristics of its cognate antigen (2, 27, 40). For instance, an IC formed by repetitive antigens can spontaneously form larger immune complexes, whereas IC composed of monomeric antigen and monoclonal antibodies cannot form larger complexes (**Figure 1A**). Several important immune receptors have evolved to preferentially activate in the presence of highly complexed antibody bound to repetitive pathogen epitopes, including complement receptor C1q, which initiates the complement cascade, and the low affinity receptor FcγRIIIa, considered to be one of the main effector FcγRs on immune cells (13). Monomeric antigen-antibody complexes have reduced capacity to induce strong immune responses because they cannot activate these pathways (41–43). An IgG fusion vaccine targeting dengue virus envelope protein domain III produced stronger B cell and T cell responses when delivered in a polymeric form compared to a monomeric form in human adenotonsillar tissue (25). Consistent with this prior work, our data shows that gD-RIC forms large complexes with strongly improved ability to bind C1q and greater overall antigenicity (**Figures 3** **–** **6**). By contrast, gD-IC forms smaller complexes with severely impaired immune receptor binding and antigenicity (**Figures 3** **–** **6**). An optimal vaccine must be cost-effective, eliciting strong responses with as few doses as possible. Only after the third dose did the IC group achieve a mean titer equivalent to a single dose of RIC (**Figure 5A**), highlighting the ability of gD-RIC to produce very strong immune responses with minimal doses without adjuvant. We have previously found that an Ebola RIC produced large heterogenous complexes (44), though with somewhat reduced C1q binding and immunogenicity compared to the present study due to incorporation of endogenous plant glycans (data to be presented elsewhere). Oligomeric antigen-antibody vaccine preparations such as RIC have now been shown to strongly enhance antigen immunogenicity using a variety of antigens, including tetantus toxin (45), Ebola glycoprotein 1 (44, 46), dengue virus envelope protein (24, 25, 47), human papillomavirus L2 (28), Zika virus envelope (29, 30), and a short 23 amino acid peptide comprising the ectodomain of influenza matrix 2 protein (data to be presented elsewhere), underscoring their broad potential to consistently and efficiently induce strong immune responses to a variety of large and small antigens.

It could be argued that the potent immunogenicity observed by gD-RIC is due in part to a mouse immune response directed against human IgG1. Notably, gD-IC and gD-RIC contain identical amounts of human IgG1. We observed modest levels of antibodies targeting the IgG1 backbone in gD-RIC, though no evidence of strong titers generated against the 6D8 epitope tag itself (**Figure S3**). To determine whether gD-RIC retains its strong immunogenicity without the human antibody backbone, we generated gD-RIC utilizing a mouse IgG2a backbone and found no statistical differences in antigen-specific antibody titers or virus neutralization compared to human gD-RIC constructs (data to be presented elsewhere). Other groups have shown that antibody complexes utilizing a mouse IgG backbone were still potent enhancers of antigen immunogenicity in mice, consistent with our observations (24). Indeed, that larger immune complexes make potent immunogens is well-established, as this feature is undesirable for intravenous antibody therapy (48). It has been known for over half a century that antibody aggregates can cause pathology such as serum sickness (49), and immune complex deposition is a driver of inflammation in diseases such as lupus erythematosus (50). Hypersensitivity reactions caused by immune complexes are a concern with all antibody therapies (51, 52). Therefore, these concerns must be addressed to ensure the safety of immune complex vaccines. Notably, antibody therapies are commonly administered with doses in the range of several grams of antibody (53). On the other hand, typical intramuscular injection of a vaccine preparation delivers 1,000-50,000 times lower doses, in the microgram range. During natural infection, immune complex formation is a necessary and desirable consequence of an effective immune response, as antibodies directed against repeated antigens, such as viral capsids, will inevitably form larger heterogeneous antibody-antigen complexes. Importantly, the immunostimulatory nature of repetitive antigens, which would generate large immune complexes upon repeated vaccination, has resulted in the success of safe and effective virus-like particle vaccines such as Gardasil (54). The RIC platform allows this enhanced immunogenicity to be conferred to antigens that otherwise do not self-assemble into larger complexes. We have not observed any toxic effects in mice across multiple RIC vaccine studies. Future studies are needed to specifically assess the safety of RIC vaccines.

**Figure 7:**
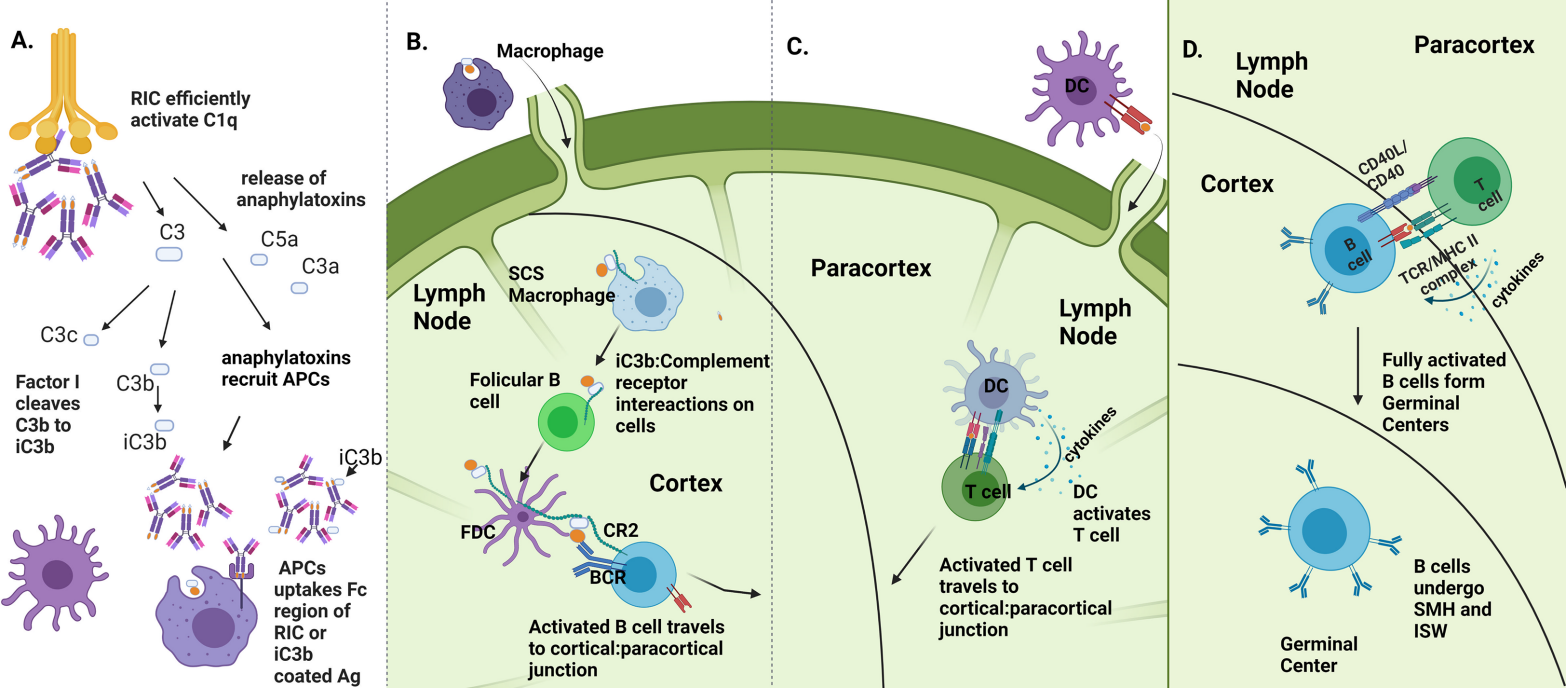
Proposed mechanisms of RIC immune enhancement. **(A)** RIC platform enhances complement activation. RICs activate C1q which in turn activates the complement cascade, causing release of C5a and C3a as cleavage products and downstream production of iC3b. Anaphylatoxin release recruits immune cells, including APCs to site of vaccination, which are able to uptake RIC antigen via iC3b:CR2 interactions and Fc:FcR interactions, made more potent via the RIC platform. **(B)** Enhanced uptake and iC3b production leads to more efficient B cell activation. Presence of iC3b is required for full B cell activation via presentation of antigen from follicular dendritic cells to naïve B cells. If B cells recognize both antigen and iC3b, B cells to migrate to cortical-paracortical junction to gain T cell help. **(C)** Enhanced DC activation and recruitment leads to more efficient T cell activation. DCs that were recruited to vaccination site travel to LN to activate T cells. Activated T cells travel to cortical: paracortical junction. **(D)** B cells gain T cell help and can effectively undergo affinity maturation. After getting help from T cells, B cells form germinal centers where they undergo somatic hypermutation and isotype switching. (*Image created using BioRender.com*).

We propose several mechanisms by which the RIC platform may enhance antigen immunogenicity, outlined in **Figure 7**. Multivalent ICs have been shown to be potent activators of complement (**Figure 4A**, (9, 25, 28–30, 45)). Although the complement system is canonically considered innate, in recent decades, the role of compliment in activating the adaptive immune system has been illustrated in several ways (12, 55, 56). Primarily, aggregated antibody complexes, like RICs, activate the complement cascade (9, 10), which results in the cleavage of complement proteins, including C5 and C3, releasing C3a and C5a as potent anaphylatoxins (57). These anaphylatoxins cause mast cell and basophil degranulation, leading to a release of vasoactive substances which increase blood flow to the site of vaccination. Multiple cell types are then activated through anaphylatoxin binding to G-protein coupled receptors on phagocytic cells that can serve as APCs, including dendritic cells and macrophages (58, 59). Activation of these APCs causes them to travel to the lymph node, leading to activation of B and T cells; it can also result in pro-inflammatory cytokine release, which successively recruits more immune cells to the vaccination site. Increases in vascular permeability in these areas can also encourage the movement of antigen-bearing APCs to draining lymph nodes, encouraging efficient elicitation of adaptive immune responses (**Figure 7A**).

Additionally, complement activation results in the production of iC3b, which, upon ligation with the CR2 receptor on follicular dendritic cells, is necessary for full B cell activation in the lymph node (60–62). In fact, a decline in circulating levels of complement can result in impaired antigen-specific antibody responses, indicating the requirement of complement presence for effective humoral responses to pathogens (63) **(** **Figure 7B**). Noting that RICs elicit high levels of antigen-specific antibodies and that CD4 T cell help in the form of cytokine and costimulatory signals is required for isotype switching and affinity maturation of antibodies to occur, we further speculate that RICs play a role in an augmented presentation of antigen to T cells and subsequent T cell activation (64). Complement proteins have been shown more recently to play a role in activating and regulating both CD4+ and CD8+ T cell responses (12). As T cells are initially activated by DCs that have traveled to the lymph node, and DCs can be recruited and activated with the help of anaphylatoxins at the vaccination site, this potential T cell regulation could occur via the increased presentation of Ag by DCs in the lymph node to T cells. Additionally, since RICs contain numerous Ig domains, FcγR on DCs can more readily take up RICs linked to antigen, allowing for enhanced internalization of antigen and increased presentation of antigen to T cells in the lymph nodes (13). Further, there is evidence that complement activation can elicit effective T cell signaling allowing for proper T cell activation (65) (**Figure 7C**). After full activation of T and B cells, both will migrate to the cortical: paracortical junction, where CD40:CD40L interactions will occur between T and B cells. This interaction not only allows for full T cell activation but also allows B cells to gain T cell help via cytokines and costimulatory molecules, necessary for them to create germinal centers and undergo somatic hypermutation and isotype switching, generating the Ag-specific responses we have illustrated following vaccination with RIC (**Figure 7D**). Future studies elucidating the precise mechanisms underlying the observed immunogenicity of RICs could lead to additional enhanced vaccination strategies and could be extended to other vaccine platforms.

The induction of neutralizing antibody titers has long been a gold standard for vaccine research, though in recent years it has been more widely appreciated that non-neutralizing antibodies are also important for protection against most viruses, including HSV (66). Compared to gD-IC, gD-RIC elicited higher total IgG titers (**Figure 5A**), higher HSV-2 and HSV-1 specific neutralization titers (**Figure 6**), and higher levels of IgG2a antibodies (**Figure 5B**). While BALB/c mice mainly produce the IgG1 antibody subclass, the antibody subclass IgG2a is an indicator of a stronger Th1 type response and these antibodies have important Fc-mediated antiviral functions (67). Due to serum limitations, neutralization studies were performed with dose 3 serum, where differences between gD-IC and gD-RIC were less pronounced than after doses 1 or 2 (**Figure 5A**). The differences between gD-IC and gD-RIC are likely reduced after dose 3 because there are diminishing returns from repeated vaccination, with the gD-RIC antibody responses in the serum eventually reaching maximal levels.

Another rationale for the design of RIC vaccines is the lack of antibody binding to the target antigen itself. Instead, RIC form by binding a defined epitope tag separated from the antigen via linker (**Figure 1B**), allowing the same universal RIC platform to function with any desired antigen (27). By contrast, a traditional IC preparation requires a new antibody to be identified and tested empirically for each new vaccine antigen. Direct antigen binding in IC may reduce the interaction of B cells specific for the already bound epitope, a phenomenon known as epitope masking. A malaria vaccine candidate was found to have limited B cell expansion after administering a third dose due to epitope masking, but increased diversification of humoral responses (68, 69). An HIV IC vaccine candidate retained similar overall antigen titers compared to vaccination with antigen alone, however epitope masking by the IC focused immune responses towards more desirable epitopes (20, 70, 71). Overall, epitope masking can be advantageous or disadvantageous depending on context. In the case of gD-IC, the soluble ectodomain of HSV-2 gD used in this study comprises 306 amino acids, spanning 17 known major epitope regions which have been extensively studied (72, 73). HSV8 binds an epitope located between amino acids 234-270 (31). Serum from immunization with HSV-2 gD was found to contain antibodies targeting a variety of linear and conformational epitopes throughout gD (74). As numerous anti-gD antibodies were able to bind even when epitopes which bind the same region as HSV8 were blocked (74), it is unlikely that epitope masking played a large role in the overall differences gD-specific titers observed between gD-IC and gD-RIC. However, after dose 3, we observed only an ~8-fold increase in mean neutralization titers with gD-RIC, despite a ~20-fold increase in mean gD-specific antibody titers (**Figures 5**, **6**). While these differences would be insignificant compared to the very large differences in total antibody titers after dose 2 (**Figure 5**), we cannot exclude the possibility that epitope masking present in gD-IC may have modestly focused antibody production towards more neutralizing epitopes compared to gD-RIC. Alternatively, gD fusion to the IgG1 heavy chain, the epitope tag fusion itself, or other factors, may have inhibited proper formation of some neutralizing epitopes in gD-RIC. These findings suggest potential room for future optimization of the RIC platform. By modifying the strength of epitope tag binding, RIC variants have been produced which were smaller, more homogenous, and more soluble, while still maintaining strong immunogenicity (29). Future studies are needed to investigate additional possible optimizations to the RIC platform, such separating the antigen with longer linkers, employing different self-multimerization strategies, or by utilizing direct antigen binding to confer advantageous epitope masking.

## 4 Experimental procedures

### 4.1 Vector construction

The construction details of pBYKEMd2-HSV8 have been previously published (75). A construct containing the HSV gD antigen fused to a 6H tag was created by digesting pBYe3R2K2Mc-BAZsE6H (30) with XhoI-SpeI to produce the expression vector fragment. The insert with the gD coding sequence was derived from a PCR-amplification of the cloned gene in pCRblunt-gD. The final construct was named pBYe3R2K2Mc-gD306-6H (referred to as “gD”).

A RIC vector containing gD linked to the humanized, 6D8 antibody C-terminus was created by PCR-amplifying pCRblunt-gD with end-tailoring primers, gD-Bam-F (5’- gggGATCCaaatatgcattagctgatcctagtc) and gD306-Spe-R (5- GCAACTAGTATGGTGTGGAGCAACATC), to add BamHI-SpeI restriction sites. The PCR product was digested BamHI-SpeI and ligated with the vector derived from pBYR11eM-h6D8ZE3 (30) to produce pBYR11eM-h6D8gD (“gD-RIC”).

### 4.2 Agroinfiltration of *Nicotiana benthamiana* leaves

Binary vectors were separately introduced into *Agrobacterium tumefaciens* EHA105 by electroporation. The resulting strains were verified by restriction digestion or PCR, grown overnight at 30°C, and used to infiltrate leaves of 5- to 6-week-old *N. benthamiana* maintained at 23-25°C. Briefly, the bacteria were pelleted by centrifugation for 5 min at 5,000g and then resuspended in infiltration buffer (10 mM 2-(N-morpholino)ethanesulfonic acid (MES), pH 5.5 and 10 mM MgSO4) to OD600=0.2, unless otherwise described. The resulting bacterial suspensions were injected by using a syringe without needle into leaves through a small puncture (76). It has been previously shown that IgG-based vaccines have enhanced immune receptor binding properties when produced in glycan-modified plants (29, 77–79), therefore transgenic plants silenced for xylosyltransferase and fucosyltransferase were employed (80). Plant tissue was harvested at 5 days post infiltration (DPI).

### 4.3 Protein extraction, expression and purification

Constructs gD-RIC, HSV8, and 6D8 were purified by protein G affinity chromatography. Agroinfiltrated leaves were blended with 1:3 (w:v) ice cold extraction buffer (25mM Tris-HCl, pH 8.0, 125mM NaCl, 3mM EDTA, 0.1% Triton X-100, 10 mg/mL sodium ascorbate, 0.3 mg/mL phenylmethylsulfonyl fluoride), stirred for 30 min at 4°C, and filtered through miracloth. To precipitate endogenous plant proteins, the pH was lowered to 4.5 with 1M phosphoric acid for 5 min while stirring on ice, then raised to 7.6 with 2M Tris base. Following centrifugation for 20 min at 16,000*g*, the clarified extract was loaded onto a Protein G column (Thermo Fisher Scientific, Waltham, MA, USA) following the manufacturer’s instructions. Purified proteins were eluted with 100mM glycine, pH 2.5, directly into collection tubes containing 1M Tris-HCl pH 8.0 to neutralize the elution buffer and stored at -80°C. Purified protein concentration was measured by A280 absorbance, ELISA, and gel quantification.

gD-His expressed from pBYe3R2K2Mc-gD6H was purified by metal affinity chromatography. Protein was extracted as described above, but without acid precipitation. The clarified extract was loaded onto a column containing TALON Metal Affinity Resin (BD Clontech, Mountain View, CA) according to the manufacturer’s instructions. The column was washed with PBS and eluted with elution buffer (PBS, 150mM imidazole, pH 7.4). Peak protein elutions were identified by SDS-PAGE, pooled, dialyzed against PBS, and stored at -80˚C. Protein concentration was measured by A280 absorbance and gel quantification.

### 4.4 SDS-PAGE and Western blot

Plant protein extracts or purified protein samples were mixed with SDS sample buffer (50 mM Tris-HCl, pH 6.8, 2% SDS, 10% glycerol, 0.02 % bromophenol blue) and separated on 4-15% stain-free polyacrylamide gels (Bio-Rad, Hercules, CA, USA). For reducing conditions, 0.5M DTT was added, and the samples were boiled for 10 min prior to loading. Polyacrylamide gels were visualized and imaged under UV light, then transferred to a PVDF membrane. The protein transferred membranes were blocked with 5% dry milk in PBST (PBS with 0.05% tween-20) overnight at 4°C and probed with goat anti-human IgG-HRP (Sigma-Aldrich, St. Louis, MO, USA diluted 1:5000 in 1% PBSTM) for IgG detection; or, probed with human HSV8 (plant-made, diluted 1:1000 from 1 mg/ml in 1% PBSTM) for gD-6H detection; or, probed with the mouse anti-gD mAb H170 (Santa Cruz Biotechnology, TX, USA, diluted 1:1000 in 1% PBSTM) for gD-RIC detection. Bound antibody was then detected with either anti-human IgG-HRP (Sigma-Aldrich, St. Louis, MO, USA, diluted 1:5000 in 1% PBSTM) for HSV8, or with anti-mouse IgG-HRP (Southern Biotech, AL, USA, diluted 1:5000 in 1% PBSTM) for H170. Bound antibodies were detected with ECL reagent (Amersham, Little Chalfont, United Kingdom).

### 4.5 ELISA

IC were prepared by incubating gD-6H and HSV8 at a 2:1 molar ratio to mimic the ratio of antigen and antibody present in gD-RIC for 2 hours at room temperature. During this time, purified RIC aliquots were thawed, and RIC and IC were serially diluted in 1% PBSTM sfor ELISA. RIC and IC were both set to a starting concentration of 10 μg/ml (including antigen and antibody weight. For immune receptor binding, 96-well medium-binding polystyrene plates (Thermo Fisher Scientific, Waltham, MA, USA) were coated with 15 μg/ml human complement C1q or human FcγRIIIa (PFA, MilliporeSigma, MA) in PBS for 1.5 hours at 37°C. For IC binding experiments, the plates were instead coated with 15 μg/ml plant-made gD-6H. The plates were washed 3 times with PBST, and then blocked with 5% dry milk in PBST for 30 minutes. After washing 3 times with PBST, for immune receptor binding experiments, purified IC or RIC were added at 10 μg/ml with a 5-fold serial dilution and were incubated for 1.5 hours at 37°C. For IC binding, HSV8 was added at initial concentration of 10 μg/ml with 5-fold serial dilutions. After washing 3 times with PBST, bound IgG was detected by incubating with a 1:500 dilution of an anti-human IgG (whole molecule) HRP-labeled probe (Sigma-Aldrich, St. Louis, MO, USA) for 1 hour at 37°C. The plates were washed 4 times with PBST, developed with TMB substrate (Thermo Fisher Scientific, Waltham, MA, USA), stopped with 1M HCl, and the absorbance was read at 450nm.

### 4.6 Sucrose gradient density centrifugation

Purified 0.5 mg samples (300 µl) of IC, RIC, mAbs, or PBS control were loaded onto discontinuous sucrose gradients consisting of 300 µl layers of 5, 10, 15, 20, and 25% sucrose in PBS in 2.0 ml microcentrifuge tubes and centrifuged at 21,000*g* for 24 h at 4°C. Nine fractions (200 µl) were collected, and the total protein content of each fraction was measured via spectrophotometry. The A280 absorbance of the PBS control fractions were subtracted from each corresponding fraction of IC or RIC. The highest absorbance value was arbitrarily assigned the value of “1” and the other fractions were calculated relative to this value. Representative results from 3 independent experiments are shown.

### 4.7 Immunization of mice and sample collection

Groups (n = 6) of female Balb/c mice, 6–7 weeks old, were immunized subcutaneously with gD-IC prepared with a 1:2 molar ratio of HSV8 to gD, or gD-RIC. An equivalent amount of 4 µg of gD was delivered per dose. The constructs were first analyzed by SDS-PAGE to detect any cleavage products, then quantified by the ImageJ software and spectroscopy to determine the percentage of gD-containing antigen. Three mice were immunized with PBS as a negative control. No adjuvant was used for any group. Doses were delivered on days 0, 28, and 56. Serum was collected by submandibular bleed as described (81) on days 0, 28, and 56, and 86. All animals were handled in accordance with the Animal Welfare Act and Arizona State University IACUC.

### 4.8 Antibody measurements

Mouse antibody titers specific for gD, 6D8 variants, or gD-RIC were measured by ELISA. Purified gD, 6D8 variants, or gD-RIC (15 μg/ml) were bound to 96-well high-binding polystyrene plates by a 1-hour incubation at 37°C (Corning Inc, Corning, NY, USA). The plates were then washed with PBST (PBS with 0.05% tween-20) and blocked with 5% nonfat dry milk in PBST. After the wells were washed with PBST, the diluted mouse sera (5-fold serial dilutions from 1:40 to 1:3,125,000 for gD, or 10-fold serial dilutions starting from 1:100 for 6D8 variant and gD-RIC ELISA) from each bleed were added and the plate incubated at 37°C for 1 hour. After washing with PBST, the mouse antibodies were detected by a 1-hour incubation with either a polyclonal goat anti-mouse IgG-horseradish peroxidase conjugate (Sigma-Aldrich, St. Louis, MO, USA) or a IgG2a horseradish peroxidase conjugate (Santa Cruz Biotechnology, Dallas, TX, USA). The plate was then developed with TMB substrate according to the manufacturer’s instructions (Thermo Fisher Scientific, Waltham, MA, USA) and the absorbance was read at 450 nm. The endpoint titers were taken as the reciprocal of the lowest dilution which produced an OD450 reading twice the background. Statistical analysis between the vaccinated groups was carried out using one-way ANOVA with Tukey’s post-test for multiple comparisons.

### 4.9 Cell culture and virus propagation

Vero cells (African green monkey kidney cells, ATCC) were cultured in a 5% CO_2_ incubator with Dulbecco’s Modified Eagle’s Media (DMEM, Cytiva), supplemented with 10% fetal bovine serum (FBS, Gibco) and 1% Penicillin-Streptomycin (Pen-Strep, Gibco). A recombinant HSV-2 strain expressing green fluorescent protein (GFP) was a kind gift from Orkide Koyuncu (UC Irvine). The HSV-1 OK14 strain, expressing a red fluorescent protein (RFP)-tagged capsid protein, was previously described (82). Viral stocks were grown on Vero cells incubated with viral media (DMEM supplemented with 2% FBS and 1% Pen-Strep). Virus stocks were harvested once significant cytopathic effect was observed and stored at -80C in 2% HEPES buffer (Gibco). Virus stocks were titered by serial dilution plaque assay, as described below.

### 4.10 Neutralization assay and plaque imaging

24-well plates were seeded 2x10^5^ Vero cells per well and incubated overnight. Mouse serum from all six individual mice was combined to a final volume of 20 µL and then diluted to a 1:5 concentration in 100 µL of viral media. This initial 1:5 dilution was then serially diluted two-fold in 100 µL volumes for an additional five dilutions. HSV-2 and HSV-1 OK14 stocks were diluted to a concentration of 500 plaque-forming units (PFU)/mL, and 100 µL (50 PFU) of this diluted working stock was mixed with each serum dilution, and incubated for 1 hour at 37°C. Vero cells were then inoculated with the serum-virus solutions for 1 hour at 37°C. Next, the inoculum was aspirated off and the cell monolayer was overlaid with 1 mL of Methocel-thickened viral medium. At 48 hours post-infection, fluorescent foci were imaged using a Nikon Ti2-E inverted widefield fluorescence microscope in the ASU Biodesign Imaging Core Facility. This microscope is equipped with a SpectraX LED light source, providing 470/24nm light for GFP excitation or 550/15nm light for RFP excitation. Fluorescence emission was captured using a Photometrics Prime95B sCMOS camera. Nikon NIS Elements software was used to produce tiled images of each entire well. All dilutions and antibody neutralizations were performed in triplicate, and mean plaques per well were used to calculate the neutralization titer. The neutralization titer (given as PRNT50) of each serum sample is defined as the reciprocal of the highest test serum dilution for which the virus infectivity is reduced by 50% when compared with the mean plaque count of the control virus with no serum added. Plaque counts for all 6 serial dilutions of serum were scored to ensure that there was a dose-response.

## Data availability statement

The original contributions presented in the study are publicly available. The gD-RIC construct information is deposited into the GenBank repository, accession OP819560.

## Ethics statement

The animal study was reviewed and approved by the Arizona State University Institutional Animal Care and Use Committee.

## Author contributions

AD, HM, IH, MB, and MP designed experiments and analyzed data. AD, AK, HM, MD, and MP constructed vectors. AD, SA, AK, and AC produced and characterized the constructs. AD performed sucrose gradient, C1q binding, and FcγRIIIa binding experiments. KL and JK performed mouse immunization and bleeds. AD and MD performed antibody titer experiments. MB performed neutralization experiments. AD and MD wrote the manuscript. AD, MP, HM, MD, and IH critically revised the MS. All authors contributed to the article and approved the submitted version.

## Supplementary material

Supplementary Figure 1 SDS-PAGE Gels and Westerns from Construct Purifications. Full gel images from each of the SDS-PAGE and western blot experiments used to compile . The lane corresponding to the image used for is indicated with an asterisk (*). **(A)** gD-RIC SDS-PAGE gel. **(B)** gD-6H SDS-PAGE gel. **(C)** HSV8 SDS-PAGE gel. **(D)** gD-RIC western blot probed with HSV8. **(E)** gD-6H western blot probed with HSV8. **(F)** gD-RIC western blot probed with anti-human IgG. **(G)** HSV8 western blot probed with anti-human IgG.

Supplementary Figure 2 SDS-PAGE Gels of Sucrose Gradient Profiles. Fractions 1-9 from sucrose gradients of 6D8, HSV8, gD-IC, and gD-RIC were separated on reducing stain free SDS-PAGE gels. Fraction 1 represents the top of the gradient with the lowest sucrose concentration, while fraction 9 represents the bottom of the gradient with the highest sucrose concentration. Note the increased proportion of gD-RIC in fractions 4-9 compared all other constructs. The leftmost lane contains protein size markers.

Supplementary Figure 3 Antibody Response to the Human IgG1 RIC Backbone ELISA measuring antibody responses using serially diluted (1:100 to 1:10,000) dose 3 serum from mice vaccinated with gD-RIC or PBS. Serum binding was measured using HL, 6D8 antibody with heavy and light chains; He, 6D8 heavy chain with epitope tag; or HLe-gD, gD-RIC containing 6D8, epitope tag, and gD. Representative results from two independent experiments are given as mean OD450 values from two samples ± standard error.

## Glossary

**Table:**

RIC	recombinant immune complex
HSV	herpes simplex virus
gD	glycoprotein D from herpes simplex virus-2
Fc	the C-terminal fragment of crystallization of IgG
Fcγ	Fc from immunoglobulin G
Fab	the antigen-binding fragment of IgG
6D8	a human IgG1 monoclonal antibody targeting a linear epitope on Ebola virus glycoprotein 1
HSV8	a human IgG1 monoclonal antibody targeting a confirmation epitope on HSV-2 gD
C1q	the first component of the classical complement pathway
ADCC	antibody-dependent cellular cytotoxicity
CDC	complement dependent cytotoxicity
FcRn	neonatal Fc receptors
DC	dendritic cell.
