# Supplementary figures and images for: A self-binding immune complex vaccine elicits strong neutralizing responses against herpes simplex virus in mice
Source: Front Immunol. 2023 May 2;14:1085911. doi: 10.3389/fimmu.2023.1085911 (PMC10186352; doi:10.3389/fimmu.2023.1085911)

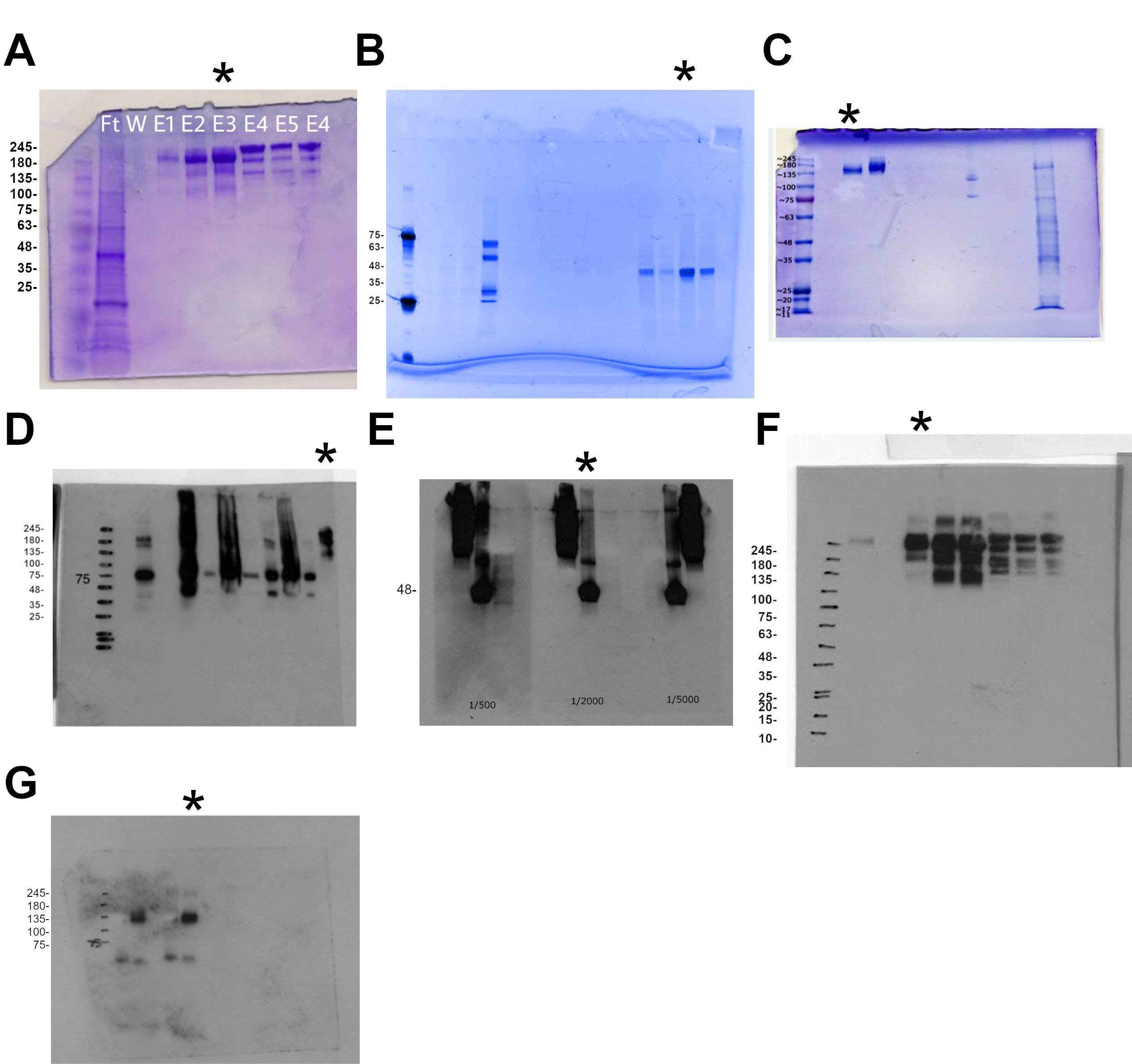

Supplement: Supplementary Figure 1 — SDS-PAGE Gels and Westerns from Construct Purifications. Full gel images from each of the SDS-PAGE and western blot experiments used to compile . The lane corresponding to the image used for is indicated with an asterisk (*). (A) gD-RIC SDS-PAGE gel. (B) gD-6H SDS-PAGE gel. (C) HSV8 SDS-PAGE gel. (D) gD-RIC western blot probed with HSV8. (E) gD-6H western blot probed with HSV8. (F) gD-RIC western blot probed with anti-human IgG. (G) HSV8 western blot probed with anti-human IgG. [file Image_1.jpeg]

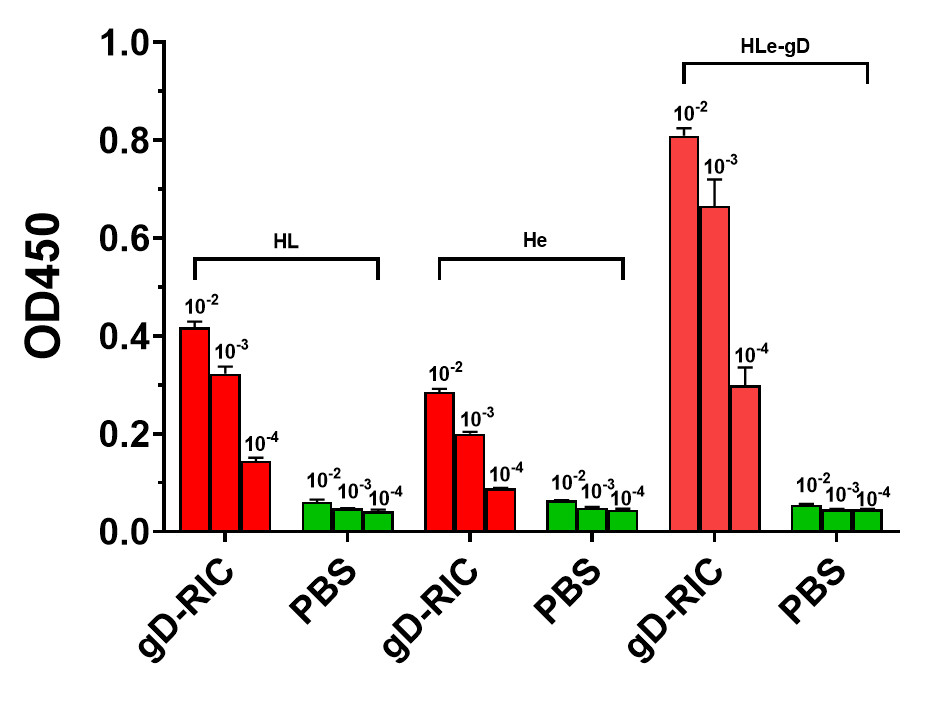

Supplement: Supplementary Figure 3 — Antibody Response to the Human IgG1 RIC Backbone ELISA measuring antibody responses using serially diluted (1:100 to 1:10,000) dose 3 serum from mice vaccinated with gD-RIC or PBS. Serum binding was measured using HL, 6D8 antibody with heavy and light chains; He, 6D8 heavy chain with epitope tag; or HLe-gD, gD-RIC containing 6D8, epitope tag, and gD. Representative results from two independent experiments are given as mean OD450 values from two samples ± standard error. [file Image_3.jpeg]
